# Integration of metabolomics, genomics, and immune phenotypes reveals the causal roles of metabolites in disease

**DOI:** 10.1186/s13059-021-02413-z

**Published:** 2021-07-06

**Authors:** Xiaojing Chu, Martin Jaeger, Joep Beumer, Olivier B. Bakker, Raul Aguirre-Gamboa, Marije Oosting, Sanne P. Smeekens, Simone Moorlag, Vera P. Mourits, Valerie A. C. M. Koeken, Charlotte de Bree, Trees Jansen, Ian T. Mathews, Khoi Dao, Mahan Najhawan, Jeramie D. Watrous, Irma Joosten, Sonia Sharma, Hans J. P. M. Koenen, Sebo Withoff, Iris H. Jonkers, Romana T. Netea-Maier, Ramnik J. Xavier, Lude Franke, Cheng-Jian Xu, Leo A. B. Joosten, Serena Sanna, Mohit Jain, Vinod Kumar, Hans Clevers, Cisca Wijmenga, Mihai G. Netea, Yang Li

**Affiliations:** 1 Department of Genetics, University of Groningen, University Medical Center Groningen, 9700 RB Groningen, the Netherlands; 2 Department of Computational Biology for Individualised Medicine, Centre for Individualised Infection Medicine, CiiM, a joint venture between the Hannover Medical School and the Helmholtz Centre for Infection Research, Hannover, Germany; 3 TWINCORE, Centre for Experimental and Clinical Infection Research, a joint venture between the Hannover Medical School and the Helmholtz Centre for Infection Research, Hannover, Germany; 4 Department of Internal Medicine and Radboud Center for Infectious Diseases, Radboud University Medical Center, 6525 HP Nijmegen, the Netherlands; 5 Oncode Institute, Hubrecht Institute-KNAW (Royal Netherlands Academy of Arts and Sciences) and University Medical Center Utrecht, 3584 CT Utrecht, the Netherlands; 6 Departments of Medicine and Pharmacology, University of California, San Diego, CA USA; 7 La Jolla Institute, La Jolla, CA USA; 8 Department of Laboratory Medicine, Laboratory for Medical Immunology, Radboud University Medical Center, 6525 GA Nijmegen, the Netherlands; 9 Department of Internal Medicine, Division of Endocrinology, Radboud University Medical Center, 6525 HP Nijmegen, the Netherlands; 10 Broad Institute of MIT and Harvard University, Cambridge, MA 02142 USA; 11 Center for Computational and Integrative Biology and Gastrointestinal Unit, Massachusetts General Hospital, Harvard School of Medicine, Boston, MA 02114 USA; 12 Oncode Institute, Princess Máxima Center for Pediatric Oncology, Heidelberglaan 25, 3584 CS Utrecht, the Netherlands; 13 Department of Immunology, University of Oslo, Oslo University Hospital, Rikshospitalet, 0372 Oslo, Norway; 14 Department for Genomics & Immunoregulation, Life and Medical Sciences Institute (LIMES), University of Bonn, 53115 Bonn, Germany

**Keywords:** Metabolomics, Genomics, Immune phenotypes, Integrative analysis

## Abstract

**Background:**

Recent studies highlight the role of metabolites in immune diseases, but it remains unknown how much of this effect is driven by genetic and non-genetic host factors.

**Result:**

We systematically investigate circulating metabolites in a cohort of 500 healthy subjects (500FG) in whom immune function and activity are deeply measured and whose genetics are profiled. Our data reveal that several major metabolic pathways, including the alanine/glutamate pathway and the arachidonic acid pathway, have a strong impact on cytokine production in response to ex vivo stimulation. We also examine the genetic regulation of metabolites associated with immune phenotypes through genome-wide association analysis and identify 29 significant loci, including eight novel independent loci. Of these, one locus (rs174584-FADS2) associated with arachidonic acid metabolism is causally associated with Crohn’s disease, suggesting it is a potential therapeutic target.

**Conclusion:**

This study provides a comprehensive map of the integration between the blood metabolome and immune phenotypes, reveals novel genetic factors that regulate blood metabolite concentrations, and proposes an integrative approach for identifying new disease treatment targets.

**Supplementary Information:**

The online version contains supplementary material available at 10.1186/s13059-021-02413-z.

## Background

A growing body of evidence has revealed that metabolites have important regulatory roles in immune system function in both health [1, 2] and disease [3–5], from vitamin D playing a role in infections and autoimmune diseases by promoting monocyte differentiation and antigen presentation [6] to modulation of cytokine responses by lipoprotein metabolites [7]. However, despite a well-recognized role for metabolism in the immune system, few large-scale studies have systematically assessed the relationship between the immune system, including functional immune measures, and the thousands of circulating blood metabolites [8, 9]. Studies to date have only assessed a limited number of metabolites that do not fully cover the diverse range of metabolic pathways that interact with immune processes. Even fewer studies have assessed the genetic effects of the metabolites that are associated with immune parameters and functions, or their potential downstream effect on immune-mediated diseases [2, 10]. A comprehensive map of metabolites and their interplay with immune function and genetic regulation would provide crucial new information to help us understand the inter-individual variation in human immune function and, consequently, the role metabolites play in disease (e.g., metabolic disease, autoimmune disease, infections, or cancer), while also identifying key interactions for mechanistic and functional understanding.

In the present study, we broadly interrogate the circulating blood metabolome and integrate 10,434 metabolite features with deep immunophenotyping from a population-based cohort (Human Functional Genomics Project, N = 500) [11–13]. We systematically associate metabolite features with eight categories of host factors consisting of baseline immune parameters (including 73 immune cell subpopulation frequencies) and immune cytokine response (91 cytokine production capacities upon stimulations). We then perform genome-wide mapping of the metabolite features associated with immune phenotypes to identify their association with immune-mediated diseases, thus highlighting causal effects and potential therapeutic targets. This work demonstrates how combining metabolite measurements with genetic data can improve our power to predict cytokine production in response to stimulations. Finally, we propose a methodological pipeline that integrates genomic, metabolomic, and immune datasets to identify novel therapeutic targets in disease.

## Results

### Comprehensive metabolomics profiling and identification of non-genetic covariables

To get a comprehensive measure of the circulating blood metabolome, three different analytical approaches were used to profile metabolites: (1) a nuclear magnetic resonance (NMR) approach (BM, Brainshake Metabolomics/Nightingale Health platform, Finland), (2) flow-injection TOF-M (GM, General Metabolomics, Boston), and (3) an integrated measurement system of NMR, gas chromatography-mass spectrometry (GC-MS) and liquid chromatography-mass spectrometry (LC-MS) (UM, untargeted metabolomics, USA) [14, 15]. BM targets 231 lipids and lipoproteins (Additional file 1: Table S1), while both GM (Additional file 2: Table S2) and UM (Additional file 3: Table S3) measure circulating metabolic features, mainly those involving amino acid, glucose, and lipid metabolism. In total, there are 231, 1589, and 8614 metabolic features measured by BM, GM, UM platforms, respectively, in the plasma of the ~ 500 Dutch participants of the 500FG cohort [11–13]. Of note, metabolic features from BM and GM have been mapped to actual metabolites, with 14 shared features (Additional file 4: Table S4), whereas a small number of the metabolic features from UM have annotations available (the “Discussion” section).

We observed substantial inter-individual variation in metabolite levels, and this variation was partly driven by host factors. For example, gender significantly influenced 63.4% of BM metabolites, 52.1% of GM metabolites, and 54.1% of UM metabolites (false discovery rate (FDR) < 0.05). Age had less influence on metabolite concentrations, with 25.1% of all metabolite features significantly associated with age, and 51.2% of these increasing with age. In total, gender contributed more variation than age to the circulating metabolites measured by GM and UM (P < 0.001, Student’s *t*-test), but this was not the case for targeted features measured using the BM platform (P = 0.172) (Additional file 5: Fig. S1a). After correction for age and gender, we also observed that body mass index (BMI) affects 5.9% of all metabolite features (FDR < 0.05, Spearman correlation analysis) (Additional file 5: Fig. S1b), with 61.9% of these positively correlated with BMI. For example, as an indicator of obesity, individuals with higher BMI also had a higher level of total fatty acids (FDR = 0.019). In addition, after correcting for age and gender, contraceptive usage affected 32.3% of metabolite features (FDR < 0.05, Spearman correlation analysis) (Additional file 5: Fig. S1b), which agrees with the known effects of contraceptive drugs on metabolism [16, 17]. We thus took the effect of contraceptive usage into account as one of the co-factors in the follow-up analysis.

### Baseline metabolites are associated with immune parameters

**Fig. 1:**
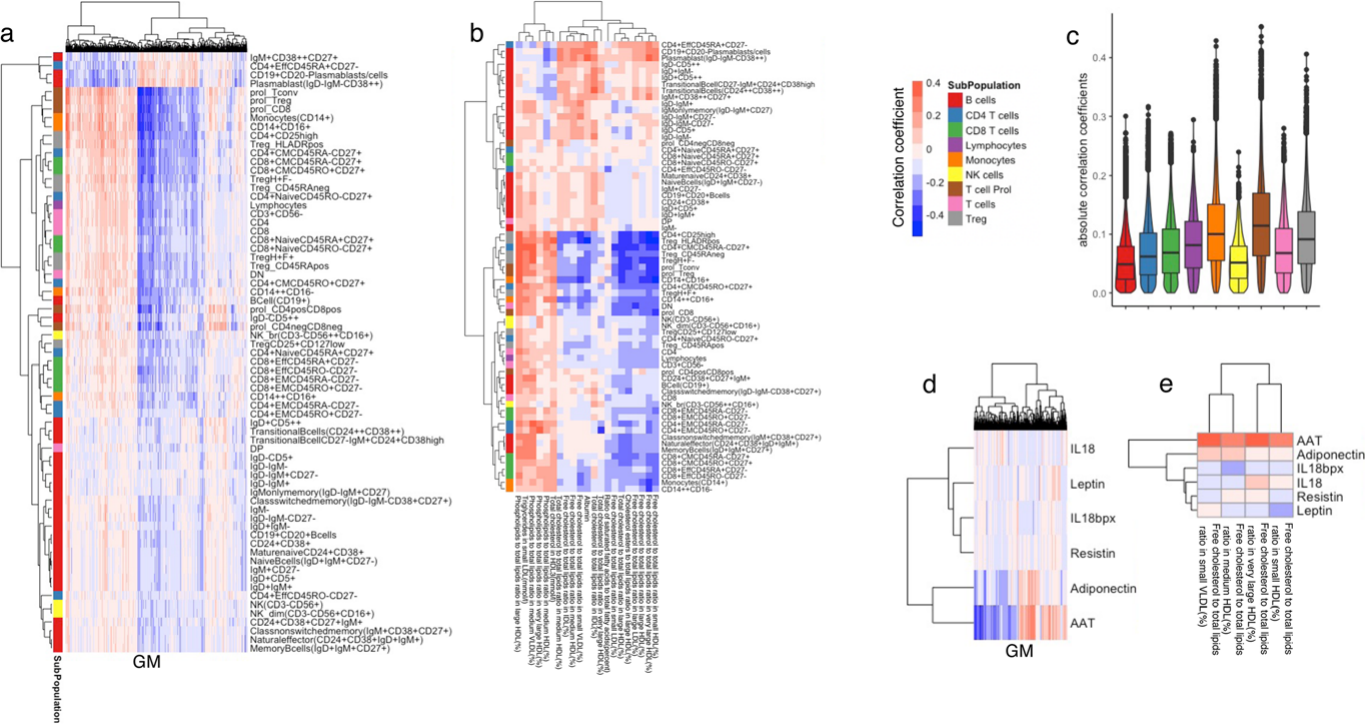
Analysis of baseline immune parameters and molecular profiling showing baseline parameters are inter-correlated. **a**, **b** Heatmap of hierarchical clustering on correlation pattern between metabolites and immune cell counts (**a** GM, **b** BM). Cell colors indicate correlation coefficients from negative (blue) to positive (red). **c** Violin and boxplots showing the absolute correlation coefficient between GM and cell counts. Colors indicate cell subpopulations. **d**, **e** Heatmap of hierarchical clustering on correlation pattern between metabolites and immune modulators (**d** GM, **e** BM). Cell colors indicate correlation coefficients from negative (blue) to positive (red)

To capture the interactions between metabolites and baseline immune parameters, we performed Spearman correlation analysis between metabolic features (GM and BM) and five categories of data including immunological modulators, immunoglobulins, platelets, cell counts, and gut microbiome, measured in the 500FG cohort [11–13, 18, 19]. After correcting the effects of age, gender, and contraceptive usage, in total, 1069 GM and 21 BM show significant correlation with at least one cell type (FDR < 0.05, Fig. 1a, b, Additional file 6: Table S5). Stronger correlations were observed between GM and T cell subpopulations (including T reg and T prol, Fig. 1c). For example, circulating free cholesterol shows a positive correlation with plasma blasts but a negative correlation with regulatory T cells.

Moreover, there are 730 GM and 4 BM showing significant association with immune modulators (including AAT and adiponectin, Fig. 1d, e, Additional file 7: Table S6). Additionally, there are 571 GM and 10 BM significantly associated with platelet traits (Additional file 8: Table S7). AAT is a serum glycoprotein that is primarily synthesized in the liver and secreted into the serum and has fatty acid binding activity [20], in line with our observation on the positive correlation between free cholesterol and AAT. Lastly, we identified in total 1 GM and 36 BM associated with immunoglobulins (FDR < 0.05, Additional file 9: Table S8) and 147 GM associated with gut microbiome traits (FDR < 0.05, Additional file 10: Table S9). In summary, our data paint an overall picture of the interactions between baseline metabolism and immune system in health.

### Metabolic pathways correlate with cytokine production upon stimulation

**Fig. 2:**
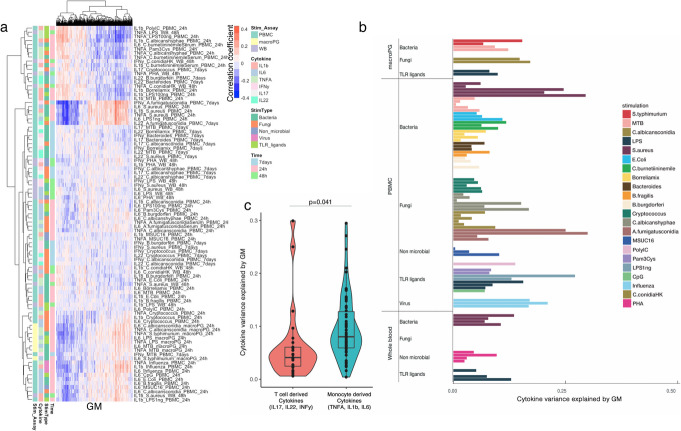
Analysis of baseline metabolites and cytokine production upon stimulation showing association and regulation of metabolites on immune response. **a** Heatmap of hierarchical clustering on correlation pattern between metabolites and cytokine production upon stimulation. **b** Cytokine variance explained by GM. The X-axis indicates explained variance represented by adjusted R squared. The Y-axis indicates stimulation types and measurement assays. Bar color shows different stimulations. **c** Violin and box plots of T cell–derived cytokine and monocyte-derived cytokine variance explained by GM. The X-axis indicates groups of cytokines grouped according to cell origins. The Y-axis indicates explained variance represented by adjusted R squared

Cytokine production capacity after stimulation is an important component of host immune defense. Previous studies have shown that genetics, environmental factors, and microbiome composition correlate with cytokine production upon human pathogen stimulation [11–13, 21]. Here we systematically characterized the extent to which baseline metabolic pathways contributed to inter-individual variation in cytokine response upon perturbation. After correcting for age, gender, and contraceptive use, we calculated the Spearman correlation between each metabolite feature and each of the 91 stimulation–cytokine pair (Additional file 11: Table S10). In total, there are 1091 and 3 metabolic features from GM and BM, respectively, showing significant association with at least one stimulation–cytokine pair (FDR < 0.05, GM: Fig. 2a, Additional file 11: Table S10). For example, there are seven metabolites: asparagine, alanine, glutamate, glutamine, oxoglutaramate, fumarate, and pyruvate, involved in alanine, aspartate, and glutamate metabolism showing significant correlation with stimulation–cytokine pairs. This result agreed with our previously published results on the individual metabolite level of glutamine [22] measured by the BM platform and the known regulatory function of these metabolites on monocyte-derived cytokines [22, 23]. Furthermore, we noticed that six metabolites involved in arachidonic acid metabolism, including phosphatidylcholine, leukotriene A4, leukotriene B4, 14,15-DHET, prostaglandin E2, and prostaglandin F2alpha showing significant correlation with stimulation–cytokine pairs. Arachidonic acid and its derived metabolites are well-known as crucial modulators of immune responses [24–26]. We next investigated how the circulating homeostatic concentrations influence and regulate immune function among eight key functional components of arachidonic acid pathway, including arachidonic acid, eicosapentaenoic acid, resolving D2, leukotriene A4, leukotriene B4, neuroprotectin D1, prostaglandin E2, and prostaglandin F2a which were measured in our data. As expected, all of them show suggestive correlation with at least one stimulation–cytokine pairs (uncorrected p values < 0.05, Spearman correlation coefficients, range −0.27–0.25). Moreover, strong positive correlations among the eight metabolites were observed (Spearman correlation coefficients, range 0.17–0.96; Additional file 12: Table S11) that mirror their known roles as reactants and products and associations at functional level [27].

Next, we systematically estimated the collective contribution of baseline metabolites to the inter-individual variation in different groups of immune response to stimulations. In general, metabolite features explain up to ~ 30% of the inter-individual variation in cytokine response upon stimulation (Fig. 2b, Additional file 5: Fig. S2a), with GM metabolites explaining significantly more inter-individual variation in monocyte-derived cytokines (IL6, IL1β, and TNFα) than T cell–derived cytokines (IL17, IL22, and IFNγ) (P = 0.04, Student’s *t*-test, Fig. 2c). This finding can be roughly replicated in metabolite features measured by the UM platform (P = 0.06, Additional file 5: Fig. S2b). These results suggest that baseline metabolism is more related to the innate immune response than to the adaptive immune response.

### Genetic factors regulate metabolites associated with immune phenotypes

**Fig. 3:**
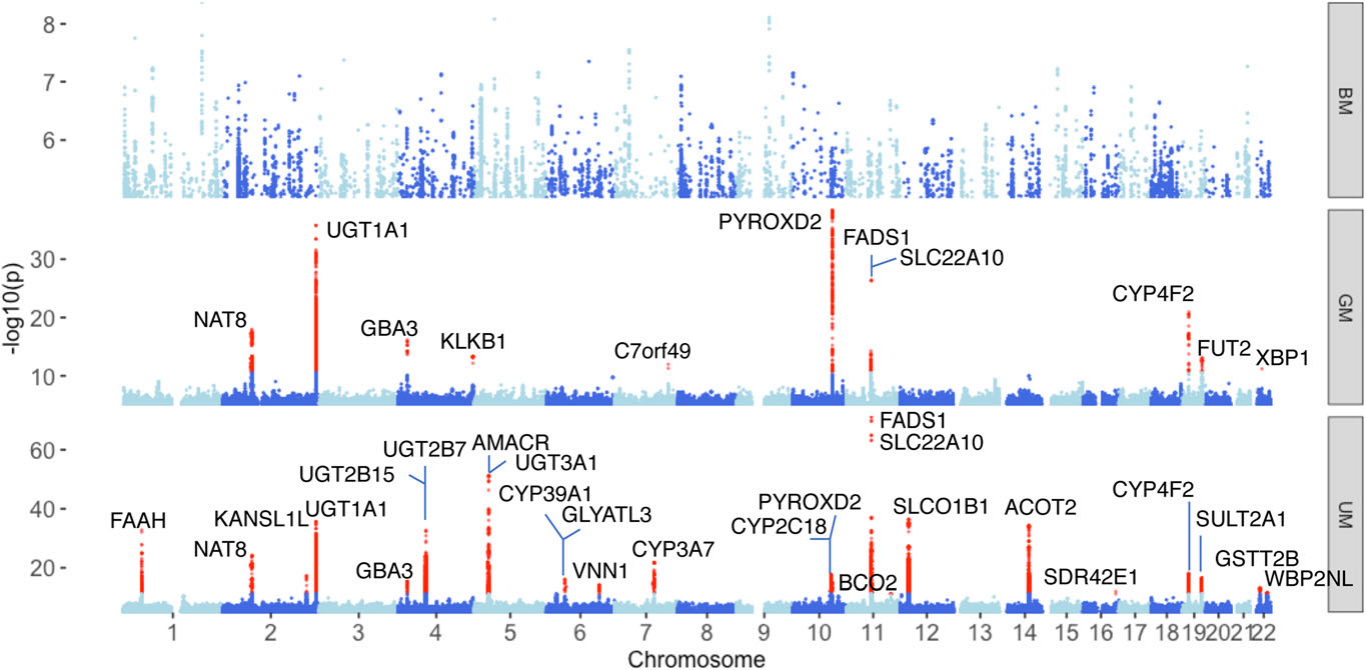
Genetic factors on baseline metabolite features. Manhattan plot of metabolite QTLs. The X-axis indicates QTL location on chromosomes. The Y-axis indicates -log10 p-values in metabolite QTL profile. Loci passing the genome-wide significant thresholds (BM: P < 2.16 × 10^−10^; GM: P < 3.15 × 10^−11^; UM: P < 5.80 × 10^−12^) are colored in red

In total, 80% of the annotated metabolite features (GM and BM) were associated with at least one immune phenotype (FDR < 0.05). We then explored the genetic determinants of them, by carrying out a genome-wide association analysis on ~ 4 million single nucleotide polymorphisms (SNPs) obtained by genotyping and imputation (see the “Methods” section). In order to acquire a more comprehensive landscape of genetic regulation on metabolism as well as an additional internal validation, we also introduced UM in this association analysis, although it has a limited annotation (the “Discussion” section). After multiple testing correction using the Bonferroni method (BM: P < 2.16 × 10^−10^; GM: P < 3.15 × 10^−11^; UM: P < 5.80 × 10^−12^), there are 11 genome-wide significant loci associated with 35 metabolic features from GM and 25 loci associated with 368 metabolic features from UM, respectively (Fig. 3, Additional files 13 and 14: Tables S12, 13). Interestingly, all of these 35 GM show a significant correlation with cytokine production upon stimulation (FDR < 0.05). Among all of these identified metabolite quantitative trait loci (mQTLs), eight were shared by GM and UM, showing internal replication, leaving 29 independent loci in total. A pathway analysis of genes mapped to 29 mQTLs shows a significant enrichment in metabolic pathways (hypergeometric test, FDR < 0.05; Additional file 5: Fig. S3), such as fatty acid, isoprenoid, and steroid acid pathways. We also noted that 22% of the genes in mQTL loci have been reported to be drug targets (Additional file 15: Table S14) [28, 29], suggesting possible pharmaceutical applications in metabolic treatment. In total, mQTLs (suggestive P < 5 × 10^−8^) explained 1.3–67.6% of the total variance in metabolites, with a median value of 8.1% based on multivariate linear regression analysis (Additional file 5: Fig. S4). These results are consistent with previous studies [30, 31] and further highlight that metabolite concentrations are under strong genetic control.

We have previously identified genetic regulation of cytokine production capacity upon stimulation in 500FG [11]. Metabolomics data measured in the same individuals gives us a unique opportunity to test if the genetic regulation of metabolites and cytokine production is shared or not. All 29 mQTLs showed nominal evidence (uncorrected P < 0.05) of association with at least one cytokine (Additional file 16: Table S15), and there was no significant difference between the effect sizes of these mQTL SNPs when we looked at monocyte-derived and T cell–derived cytokines (P = 0.20, Student’s *t-*test). This suggests that the stronger relationship we observe between baseline metabolism and innate immune response, as compared to adaptive immune response, is independent of genetics.

### Novel mQTLs reveal metabolite-associated genes

Among the 29 genome-wide significant mQTLs, eight were novel, while the remaining 21 had been identified in previous studies [10, 30–35] (Additional file 13: Table S12). For example, the mQTL of one of the unknown metabolite features, (un_407.327) with m/z = 407.327, is located in an intronic region of *VNN1* (Additional file 5: Fig. S5). VNN1 is a pantetheine hydrolase that catalyzes the hydrolysis of pantetheine to cysteamine and pantothenic acid (vitamin B5), which are both potent antioxidants. Pantothenic acid is then reused for coenzyme A biosynthesis [36]. The top SNP of the *VNN1* locus, rs2050154, has an eQTL effect on vanin-1 expression levels in blood (eqtlGen [37], P = 3.2717 × 10^−310^, GTEx [38], P = 3.6 × 10^−47^). These results suggest a potential genetic regulatory role on circulating metabolites through modulation of *VNN1* expression levels. Interestingly, the *VNN1* gene has been found to be involved in asthma corticosteroid treatment [39] and to be regulated at the protein level by pro-inflammatory cytokines [40]. Interestingly, un_407.327 was found to be suggestively associated to IL17, IL1b, and IFNy in response to Bacteroides, *S. aureus*, and LPS (nominal P < 0.05, Additional file 17: Table S16). This highlights the potential link between pathways that influence baseline metabolite levels and immune responses upon stimulation, an effect that might ultimately link to immune disease.

### mQTLs enriched in non-synonymous variants

**Fig. 4:**
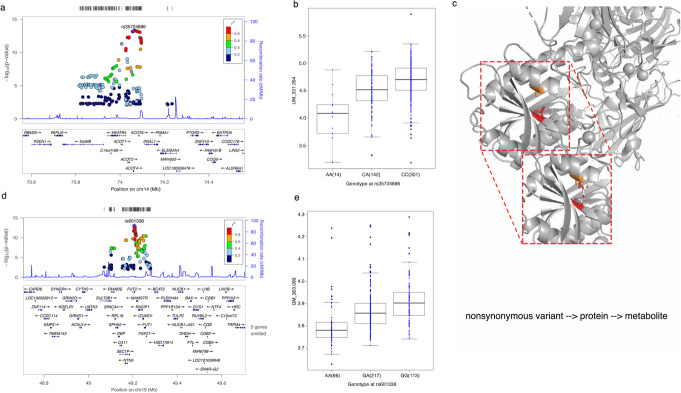
Non-synonymous metabolite QTLs associated with metabolite features in healthy volunteers. **a** Locus zoom plot showing a non-synonymous mQTL rs35724886 located on chromosome 14. **b** Box plot of the top metabolite feature (m/z 331.264) associated with genotype at rs35724886. **c** Structural visualization of ACOT4. Sticks indicate amino acid residues involved. Amino acid change induced by mQTL (red) is predicted to clash with the neighbor amino acid (orange) with Van der Waals overlap indicated by red disks. **d** Locus zoom plot showing a non-synonymous mQTL rs601338 located on chromosome 19. **e** Box plot of the top metabolite feature (m/z 363.089) associated with genotype at rs601338

We next explored the function of the genetic variants within 29 genome-wide significant mQTLs using a permutation-based method (see the “Methods” section), which revealed that mQTLs are enriched in exonic regions and 3′ UTR (P < 0.001). Among the 62 exonic SNPs in the 29 mQTL regions, 38 were non-synonymous or stop gain/loss (Additional files 18 and 19: Tables S17, 18), and these were significantly over-represented (P < 0.001). We then evaluated their biological consequences using two computational prediction tools, SIFT [41] and Polyphen2 [42]. Of the 38 non-synonymous mutations, four were predicted to have a deleterious effect on protein function (Additional file 20: Table S19). rs35724886 (minor allele frequency (MAF) = 0.18 in European populations (EUR)), for example, regulates the abundance of several metabolite features and is one of the deleterious non-synonymous variants within 29 mQTLs identified for a metabolic enzyme, Acyl-CoA thioesterase 4 (ACOT4) (Fig. 4a, b). ACOT4 is known to transform medium- or long-chain fatty acids combined with CoA into CoA and free fatty acid. To explore this further, we carried out a computational prediction analysis for the protein structure of ACOT4 for both wild and mutant types. As shown in Fig. 4, the associated Acyl-CoA thioesterase 4 deficiency rs35724886 (p. Ala187Asp) is located in a β-sheet domain, which likely leads to steric clashes with neighboring residues (colored orange in the figure) (Fig. 4c). This probably causes a reduction in function and a subsequent decrease in serum-free fatty acids. Another example of a non-synonymous variant with deleterious effect is rs601338 (MAF = 0.43 in EUR), which we observed to be significantly associated with a non-target metabolite (m/z 363.089) (Fig. 4d, e) and leads to a stop gain of transcription of *FUT2*. rs601338 influences expression levels of *FUT2* in the small intestine (P = 1.3 × 10^−7^) and stomach (P = 7.6 × 10^−25^) in the GTEx dataset [38]. Altogether, these results suggest that deleterious effects arising from non-synonymous and stop gain/loss variants in exonic regions could be one of the mechanisms behind genetic regulation of metabolite levels in the blood.

### The arachidonic acid mQTL locus shows functional and immunological relevance in disease

**Fig. 5:**
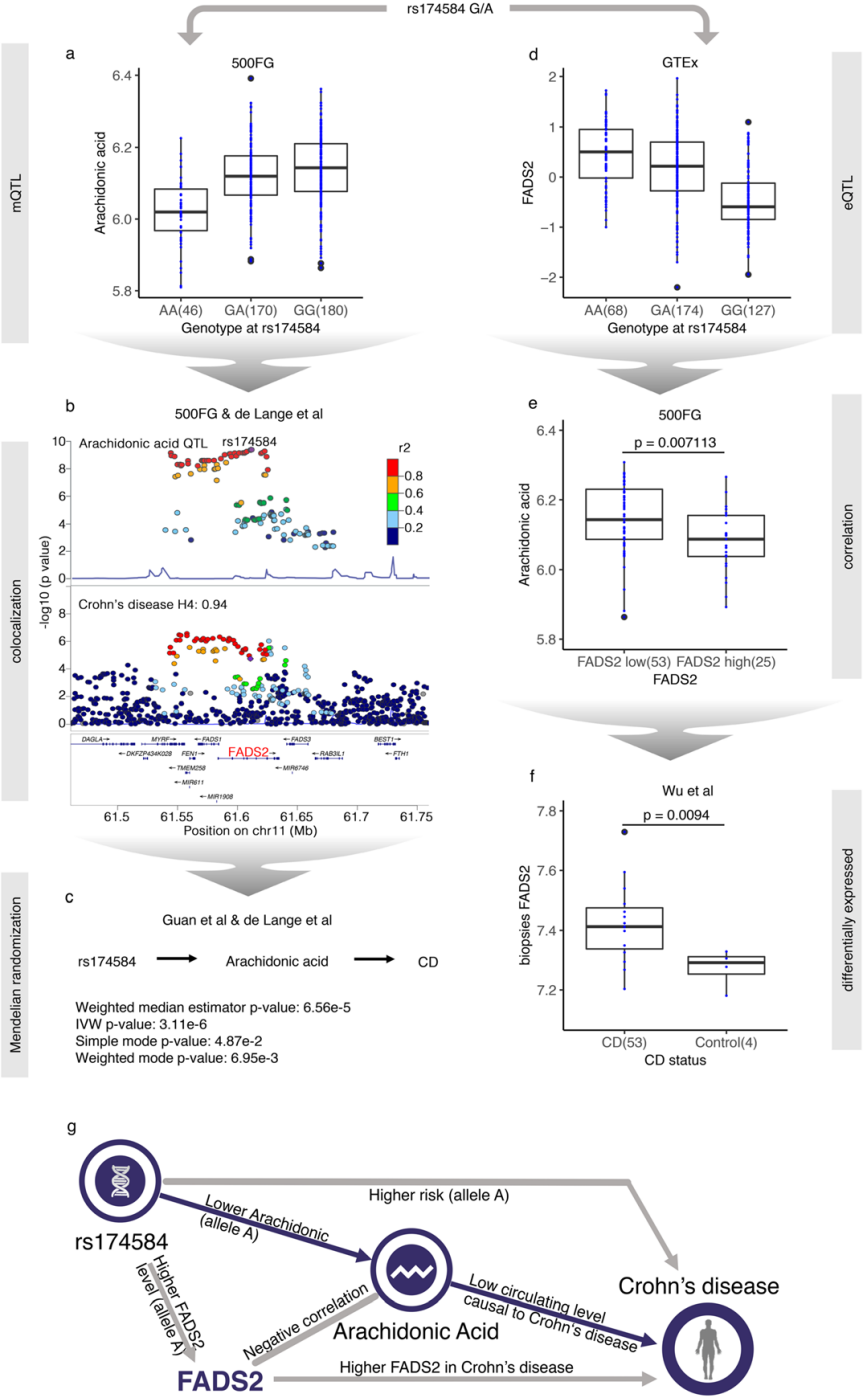
Arachidonic acid has a causal effect on Crohn’s disease through an mQTL locus. **a** Box plot of arachidonic acid level with genotype at rs174584. **b** Locus zoom plots of arachidonic acid QTL profile and Crohn’s disease GWAS profiles showing colocalization through the rs174584 locus. **c** Mendelian randomization results. **d** Box plot of blood *FADS2* expression level with genotype at rs174584. **e** Box plot showing arachidonic acid level changes with *FADS2* levels in the blood. **f** Box plot of *FADS2* expression level in Crohn’s disease (CD) biopsies versus control **g** A graphic summary of the regulation network of mQTL (rs174584-*FADS2*)

We next applied a colocalization analysis [43] between all suggestive mQTLs passing genome-wide significant threshold 5 × 10^−8^ and ten autoimmune diseases such as inflammatory bowel disease as well as other diseases such as Alzheimer’s disease and type 2 diabetes (Additional file 21: Table S20). Five GM QTLs were found to be colocalized with at least one disease trait (Additional file 21: Table S20). Among them, a mQTL suggestively associating with arachidonic acid on Chr 11 (P = 4.15 × 10^−10^, Fig. 5a) has been previously associated to Crohn’s disease (P = 1.83 × 10^−5^) [44]. It has also been associated to neutrophil count (P = 2.18 × 10^−9^) and monocyte CD14+ proportions (P = 4.72 × 10^−13^) in the blood [45], and these two cell subpopulations have been reported to be involved in the pathogenesis of Crohn’s disease [46].

Colocalization analysis [43] upon arachidonic acid mQTL and the latest Crohn’s disease genome-wide association study (GWAS) profile [44] strongly supported the hypothesis that arachidonic acid shares a common genetic variant with Crohn’s disease (posterior probability = 0.94, Fig. 5b). We then applied the Mendelian randomization [47] (MR) method to test the causal effect of arachidonic acid on Crohn’s disease using public GWAS summary statistics for both traits [44, 48]. Using eight independent SNPs (R^2^ < 0.01) as instruments, the results of four commonly used MR methods—weighted median estimator [49], inverse-variance weighted [50], and mode-based estimator in both simple mode and weighted mode [51]—consistently showed that the decrease in circulating arachidonic acid level had a causal effect on Crohn’s disease (P = 6.56 × 10^−5^, 3.11 × 10^−6^, 4.87 × 10^−2^, and 6.95 × 10^−3^, respectively; effect sizes = −0.06, −0.07, −0.07, and −0.06, respectively; Fig. 5c, Additional file 5: Fig. S6a). There was no evidence of heterogeneity between causal effects derived from these eight SNPs (Cochran’s Q, P = 0.17). Interestingly, the arachidonic acid level has been found to be significantly lower in the blood of Crohn’s disease patients compared to healthy controls [52, 53], which supports a causal relationship between blood arachidonic acid level and Crohn’s disease.

Next, we integrated transcriptome data to explore the regulatory mechanism linking the SNPs to Crohn’s disease. Previous findings have indicated that genetic variants in the *FADS1/FADS2* locus were associated to fatty acid metabolism, including the arachidonic acid pathway [54]. We find that rs174584 shows a regulatory effect on the expression of *FADS2* in blood in the GTEx [38] dataset, with allele A increasing *FADS2* expression levels (P = 4.43 × 10^−21^, Fig. 5d). In addition, *FADS2* has been shown to have a desaturase function in the transformation of arachidonic acid pathway metabolites [55]. This was confirmed by our RNA-seq and metabolomics data from 89 samples from the 500FG cohort where individuals were divided into two groups according to individual *FADS2* expression value compared to mean *FADS2* expression value. Individuals with higher expression levels of *FADS2* showed significantly lower levels of circulating arachidonic acid (P = 0.007, Student’s *t*-test; Fig. 5e). This is consistent with previous work that reported *FADS2* to be associated with Crohn’s disease [56] and with the significantly increased expression of *FADS2* (P = 0.009, Student’s *t*-test; Fig. 5f) that we observed in endoscopic pinch biopsies of Crohn’s disease patients compared to healthy donors using a previously published dataset [57].

We then investigated if *FADS2* plays a role in regulating immune functions using the 500FG datasets. Notably, the gene expression level of *FADS2* shows a positive correlation with TNFα production stimulated by *Aspergillus fumigatus* conidia and *C. albicans* (Additional file 5: Fig. S6b), which supports the immunological relevance of *FADS2*. To experimentally replicate these correlations, we stimulated peripheral blood mononuclear cells from three healthy donors with heat-killed *Candida* (*Candida* HK) and measured the TNFα level after 24 h. Compared to the control group, TNFα production decreased in the *FADS2*-inhibited group after 24-h stimulation with *Candida* HK, which suggests that *FADS2* has a promoting effect on immune response (Additional file 5: Fig. S6c). Moreover, to assess the role of *FADS2* for intestinal homeostasis, we performed repeated attempts to develop intestinal organoids on a *FADS2*-deficient background. However, in all these experiments, both homozygous and heterozygous *FADS2* clones failed to develop intestinal organoids. These results suggest that *FADS2* is important for intestinal development and/or repair, the mechanisms through which it could impact intestinal pathology (Additional file 5: Fig. S6d). Taken together, our data suggest that *FADS2* could have a pathogenic role, as TNFα is the most common treatment target in Crohn’s disease [58].

In summary, our results depict a comprehensive regulatory network, from genomic variant to disease through regulation of gene expression, metabolite levels, and immune function, based on multi-omics data from the 500FG cohort, public databases, literature, and ex vivo experiments (Fig. 5g).

### mQTLs are enriched in genetic risk factors for pro-inflammatory traits

In addition to the arachidonic acid QTL that colocalized with Crohn’s disease, the *FUT2* locus led by non-synonymous variant rs601338 also showed colocalization with three immune-mediated diseases: Celiac disease [59] (Coloc analysis H4: 0.7, H3: 0.004), Crohn’s disease [44] (Coloc analysis H4: 0.98), and type 1 diabetes (Coloc analysis H4: 0.99) [60]. This suggests that the non-target metabolite m/z 363.0888 has immunomodulatory capabilities through *FUT2* and thus has potential effects in Celiac, Crohn’s disease, and type 1 diabetes (Additional file 5: Fig. S7a). To systematically investigate the overall metabolic association of different diseases, we overlapped our mQTLs under different thresholds (P < 4.8 × 10^−12^ (5 × 10^−8^/(231BM + 1589GM + 8614UM)), 5 × 10^−8^, 1 × 10^−6^, and 1 × 10^−5^) with GWAS catalog SNPs known to influence disease susceptibility (Additional file 5: Fig. S7b). As expected, mQTLs identified in our study are significantly overlapped with other public metabolic profiles using height as reference (P = 1.52 × 10^−132^, Fisher’s exact test). We also observed significant genetic overlap between mQTLs and auto-inflammatory traits (P = 3.54 × 10^−31^), blood-related phenotypes (hematocrit and mean platelet volume, P = 9.72 × 10^−15^), heart rate (P = 1.19 × 10^−44^), and type 2 diabetes as represented by fasting glucose-related traits (P = 1.75 × 10^−35^). These enrichment results are also consistent at multiple mQTL thresholds (P < 4.8 × 10^−12^, 5 × 10^−8^, 1 × 10^−6^, and 1 × 10^−5^; Additional file 5: Fig. S7b**)**. These genetic regulatory components shared between metabolites and diseases suggest that metabolism plays a functional role in complex phenotypes in humans.

### Metabolite features have predictive power for cytokine production upon stimulation

To assess the extent to which metabolites explain inter-individual variations in cytokine production (in addition to genetic factors), we calculated the cumulative cytokine variance explained by all baseline features. While the largest effect still came from genetic factors, metabolites had an additional contribution (0.048 in average) to the inter-individual variation in cytokine response (Additional file 5: Fig. S8).

**Fig. 6:**
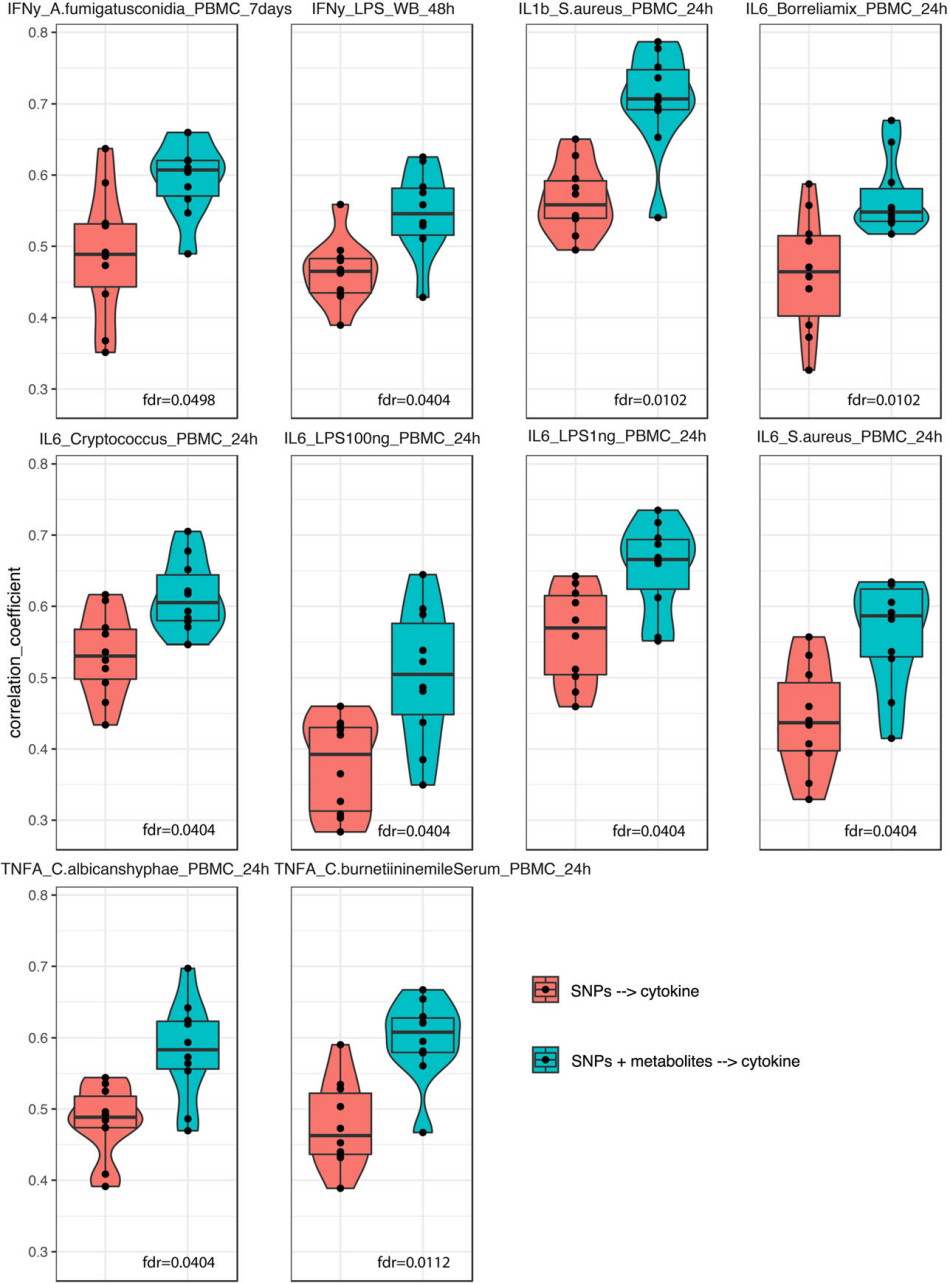
Improvements in prediction after adding metabolite information on top of genetics. Violin and box plots of Spearman correlation coefficients between predicted values and measured values in testing sets

One of our previous studies [21] showed that genetic variants moderately predict cytokine production upon stimulation. Here we tested if baseline metabolite concentrations can improve predictive power. We first constructed a prediction model for cytokine production using genetic variants identified in a previous study [11] and metabolite features measured in the 500FG cohort. We then compared our model’s prediction performance with that of the earlier SNP-only prediction model. To obtain a robust estimate of prediction performance, we applied a cross-validation strategy by randomly splitting the data into training and validation sets multiple times. What we observed was a significant improvement (FDR < 0.05, Student’s *t-*test) in prediction performance after adding metabolite data to the model, mostly coming from monocyte-derived cytokine production upon stimulation (IL1β, TNFα, and IL6). This suggests that baseline metabolites have effects on cytokine production that are independent and in addition to genetic variation (Fig. 6).

## Discussion

In this study, we have generated a comprehensive map of blood metabolites, immune phenotypes, and their genetic basis that reveals novel genetic factors that regulate blood metabolite concentrations. This work highlights the importance of baseline metabolites in immune function and immune diseases.

Taking advantage of the uniqueness of the multi-omics data available for the 500FG cohort, we systematically investigated the associations between metabolites and other immune phenotypes. We present several metabolic pathways associated with immune functions, such as the alanine and arachidonic acid pathways, and report exact associations. These findings provide both an important resource and experimental evidence for immunological and metabolic studies. The metabolites and genes we have identified are potential targets for immune-related disease studies.

Our results also suggest that baseline metabolites have a stronger impact on the inter-individual variation of monocyte-derived cytokines (TNFα, IL1β, IL6) than on T cell–derived cytokines (IL17, IL22, INFγ), which implies that baseline metabolism is more involved in the innate immune response than in the adaptive response. Innate immune cells are wired to respond to the environment [61], and subsequently activate adaptive immune cells such as lymphocytes. The classical activation of adaptive immune cells depends on stimulatory signals from antigen-presenting cells (antigens, co-stimulatory molecules, and cytokines). It is therefore likely that environmental clues, such as metabolites, are mainly sensed by cells of the innate immune system, and the release of pro-inflammatory cytokines by innate immune cells is how the regulatory system subsequently integrates innate and adaptive immune responses. This concept is supported by our observation that cytokines released from innate immune cells are more strongly influenced by circulating metabolites.

Thus far, several GWAS studies have focused on metabolite measurements using a single analytical approach. In contrast, this study presents comprehensive measurements from three different platforms that map > 10,000 metabolic features covering glucose, lipid, amino acid, and lipoprotein metabolism (among others). We took advantage of the accurate annotation of targeted measurement (BM) in functional interpretation and of the high-throughput and unbiased measurement of untargeted approaches (GM and UM) in genetic factor identification. Even with the relatively limited sample size of our cohort, we were able to replicate 21 (out of 29) previously detected mQTLs and identify eight novel genomic loci (such as the *VNN1* locus) with regulatory effects on circulating metabolite concentration. Our results can be accessed through an online browser (https://500 fg-hfgp.bbmri.nl) for future studies. We further highlight that deleterious effects arising from non-synonymous variants in exonic regions could be one of the mechanisms behind the genetic regulation of metabolite levels in the blood.

Our findings also uncovered the role of specific metabolites in the etiology of several immune-related diseases. For example, lower circulating arachidonic acid was found to be causally linked to Crohn’s disease. In agreement with previous studies in which arachidonic acid and *FADS2* were found to be related to Crohn’s disease [52, 53], our data from a population-based cohort systematically revealed (1) the association between *FADS2* and arachidonic acid, (2) the association between the arachidonic acid pathway and immune phenotypes, and (3) the association between *FADS2* and immune phenotypes (i.e., TNFα). Furthermore, by integrating our data with other public data, we confirmed the association between *FADS2* and Crohn’s disease and the association between arachidonic acid and Crohn’s disease. Since the gut is the more-relevant tissue compared to blood (where we measured arachidonic acid), we used a gut-specific organoid validation to provide further evidence supporting *FADS2* as a key driver of Crohn’s disease and highlight how integration approaches can be used to infer novel disease-relevant markers using multi-omics data. Interestingly, 54 genes within the 29 mQTL loci we identified have been reported to be candidates for metabolic drug targets (e.g., *CYP4V2*) in relevant immune diseases, and further validation of their potential as therapeutic targets is warranted.

This study analyzed a very complex set of phenotypes, and we must therefore acknowledge possible confounders and study limitations. Firstly, samples were collected in a standardized time-frame (morning) to limit possible bias, but were taken in different months of the year, which might have introduced unwanted variation caused by season. However, we observed no clear batch or month effect in the metabolic measurements. Secondly, non-fasting blood samples were drawn in the 500FG cohort, which meant that diet could have impacted blood metabolism [62, 63]. However, even with the added variability induced by diet, our analysis still had sufficient power to detect a number of novel genetic associations. Furthermore, this study used the largest cohort to date to examine interactions between metabolism and immune parameters/function. We acknowledge that our sample size was limited for the detection of weak or moderate effects and an experiment with a larger sample size will be needed for further interpretation. For some of the suggestive hits with nominal significance, we explored their potential biological mechanism through integration of a publicly available database. Lastly, the Bonferroni correction threshold we chose in the mQTL analysis, which was based on the assumption that metabolic features are independent signals, is very conservative. This could have limited our power to detect mQTLs. At the same time, although we acquired more genetic loci by introducing the unannotated UM in mQTL identification, the functional interpretation of these loci was challenging due to the lack of full accurate annotation. Improvements can, of course, be made in the future, e.g., accurate annotation of the metabolic features derived from mass spectrometry–based platforms (especially UM) would help in evaluating the precise overlap between metabolic platforms to better access metabolic pathways.

## Conclusions

This study provides insights into how genetic differences impact metabolite levels, shape immune responses, and impact disease risk, information important for future biomedical and pharmaceutical targeting. In future studies, longitudinal measurements are needed to acquire more consistent and accurate circulating metabolite levels. In addition, single-cell RNA-sequencing technology could be used to study cell type–specific effects and uncover the interaction between genes and metabolites in immune-related diseases.

## Methods

### Study cohort

Analysis was mainly performed in the 500FG cohort (part of the Human Functional Genomics Project) which consists of 534 healthy individuals (237 males and 296 females) of Caucasian origin. Their ages range from 18 to 75, with the majority (421 individuals) being 30 years old or younger. Volunteers with a mixed or other genetic background were excluded as were volunteers diagnosed with long-term diseases. Within this cohort, immune cell counts, cytokine production upon stimulations, platelets, globulins, and gut microbiome were measured. More detailed information can be found in previous publications [11, 12, 18, 21].

### Metabolomics measurement

Serum metabolite levels were measured by three different technical platforms (BM, UM, and GM) in 500 healthy Dutch individuals. BM indicates samples measured on the Brainshake Metabolomics/Nightingale Health metabolic platform. These samples were processed following the automated standard protocol provided by Nightingale’s technology (Finland), and blood metabolites were quantified in absolute concentrations (e.g., mmol/L) and percentages using nuclear magnetic resonance (NMR) spectroscopy. On the UM platform (Creative Dynamics Inc, NY, USA), which mainly focuses on lipid metabolism, metabolites identified as m/z were measured in a large scale using a measurement system that integrates NMR, GC-MS, and LC-MS. Details can be found in the references [14, 15]. GM was measured and annotated by general metabolomics (Boston, USA) using flow injection-time-of-flight mass (flow-injection TOF-M) spectrometry.

Principal component analysis (PCA) was done with log10-transformed values. Sample values > 4 standard deviations from the mean value of PC1 and PC2 were considered as outliers, leading to the removal of one sample in the UM data.

We checked the normal distribution of metabolite levels in the data from each platform using the Shapiro test. To achieve normality and consistency for QTL mapping, we log-transformed the metabolite data.

### Genotype data

Genotype data from ~ 500 healthy Dutch individuals was measured using Illumina humanOmniExpress Exome-8v1.0 SNP chip Calling by Opticall 7.0 [64] with default settings. Samples with a call rate < 0.99 were removed in further analysis, and HWE = 1× 10^−4^ and MAF = 0.05 were used for SNP quality control. After removing 17 ethnic outliers identified by multidimensional scaling, genotype data was imputed taking Genome of the Netherlands as reference. For further description, see the reference [11].

### Immune phenotypes

Other baseline immune parameters, including 73 immune cell subpopulation frequencies, cytokine production response to 19 stimulations (91 different cytokine-stimulus pairs), modulators, immunoglobulins, and platelets, were measured in 500FG. Details can be found in the references [11, 18, 21].

### Transcriptome data

To measure gene expression data, RNA sequencing was performed on a subset of 89 samples from 500FG using Illumina Hiseq 2000 platform as previously described [11, 18, 21].

### Gut microbiome

Stool samples were collected 1 day prior to or on the day of blood collection. DNA of the gut microbiome was extracted and sequenced using the Illumina HiSeq 2000 platform. Taxonomic profiles were inferred with MetaPhlAn 2.2, and functional profiling was performed using HUMAnN2. This yielded 219 species and 639 MetaCyc pathways, as described in the reference [12].

## Statistical methods

### Data pre-filtering

We intersected genotyped samples with samples from metabolite profile data and end up with 340 overlapping samples for BM QTL analysis, 397 for GM, and 458 for UM.

### Correlation analysis

Spearman correlation analysis was performed between metabolites and other types of data. Unsupervised hierarchical clustering using the “complete” approach based on “Euclidean” distance of Spearman correlation coefficients is shown as a heatmap created using the R package pheatmap.

### Estimation of explained variance

To estimate the cytokine variance explained by metabolites and other immune parameters, we first filtered the features based on their Spearman correlation p-values, keeping only features passing specific thresholds (0.001 for metabolites, 0.05 for other features) for further analysis. Potential confounder effects were then regressed out, and after removing collinearity, features were used in a multivariate linear model to estimate the proportion of variance explained indicated by total model-adjusted R^2^. Details of the method can be found in a previous paper [21].

### mQTL mapping and annotation

mQTL mapping was done with the R Package Matrix-eQTL, taking age, gender, contraceptive usage, and cell population abundance as covariates in the linear model. A p-value < 4.8 × 10^−12^ was considered to be genome-wide significant. SNPs with linkage disequilibrium > 0.1 were identified as single genomic loci.

To determine the accumulative effect of genetic factors on baseline metabolites, we applied a multivariate linear model to evaluate the metabolite variance explained by genetics after regressing out the contributions of age, gender, and contraceptive drug usage. Of the 1553 metabolites with suggestive mQTLs, 752 were measured in all the genotyped samples with no missing values. Total model-adjusted R^2^ was considered as the proportion of explained variance.

Associated variants were annotated using Annovar [65], webgestalt [66], and FUMA [67] for chromosome locations, enriched pathways, exonic SNP function prediction, and independent loci identification. A 10-kb window was used to identify genes physically located within the loci. Pymol (The PyMOL Molecular Graphics System, version 1.7.6.0, Schrödinger) was used to show protein structure changes by non-synonymous mQTLs. An online tool, MetaboAnalyst 4.0 [68], was used for metabolite pathway analysis. Functional/structural enrichment analysis on SNPs was done using a permutation-based approach. We performed functional/structure annotation on 1000 permuted sets of variants showing no significant association with any metabolite feature. We randomly selected same-sized SNPs for each permuted set and ended up with a null distribution for each functional class. We then compared the null distribution with the functional annotation of the mQTLs.

### Colocalization analysis

We performed colocalization analysis [43] to look at the overlapping profile between mQTLs and disease GWAS using the R package “coloc.” Public GWAS summary statistics performed in the European population were collected as reference.

### Mendelian randomization

MR [47] is a statistical method for identifying causality between exposure and outcome (arachidonic acid level and Crohn’s disease here) using genetic variants as instruments. We selected 5 × 10^−8^ as the threshold for arachidonic acid GWAS summary statistics, and only independent SNPs (r^2^ < 0.01) were kept for MR analysis using the R package TwoSampleMR [69]. Four common analytical methods, weighted median, inverse-variance weighted, simple mode, and weighted mode regression [49–51], were applied to detect the causal effect.

### Establishment of colon organoids

Tissues from a human colon were obtained from the UMC Utrecht with informed consent of the patient. The normal, non-transformed, mucosa was obtained from a patient with colon adenocarcinoma that was resected. The study was approved by the UMC Utrecht (Utrecht, the Netherlands) ethical committee and was in accordance with the Declaration of Helsinki and according to Dutch law. This study is compliant with all relevant ethical regulations regarding research involving human participants. Human intestinal cells were isolated, processed, and cultured as described previously [70].

### Generation of FADS2 knockout and genotyping

To generate *FADS2* knockout organoids, gRNAs were selected using the Atum website and cloned in the Cas9-EGFP vector (addgene plasmid #48138) following the protocol described before [71]. gRNAs used in this study were:

**Table:** 

FADS2_guide1_forward	**CACCG**CCAGACTTACGTTCTTGCCG
FADS2_guide1_reverse	**AAAC**CGGCAAGAACGTAAGTCTGG**C**
FADS2_guide2_forward	**CACCG**CTTGTCCACAAATTCGTCAT
FADS2_guide2_reverse	**AAAC**ATGACGAATTTGTGGACAAG**C**

Human colon organoids were transfected using these gRNAs cloned into the Cas9-EGFP vector, utilizing electroporation following a previously established protocol [72]. One week after transfection, cells were sorted for EGFP positivity using a FACS-ARIA (BD Biosciences). Wnt-surrogate (0.15 nM, U-Protein Expression) and Rho kinase inhibitor (10 μM, Calbiochem) were added to the culture medium up to 1 week after sorting to enhance single cell outgrowth. Organoids grown from FADS2gRNA/Cas9-EGFP transfected cells were genotyped for one of the two loci to establish frameshift mutations. Primers used for genotyping were:

**Table:** 

FADS2_guide1_forward	AAGGCACTCAGCTCACGAG
FADS2_guide1_reverse	TTTCTCAAAGAGGTGCCCCG
FADS2_guide2_forward	GGCTGAGGACATGAACCTGT
FADS2_guide2_reverse	AATTAGTCAGGCATGGTGGC

### GWAS enrichment analysis

GWAS SNPs were collected from the National Human Genome Research Institute GWAS catalog grouped based on phenotype association [73] including cancer, immune-mediated diseases, infectious disease, blood-related traits, heart-related traits, metabolic traits, type 2 diabetes–related traits, and height. We considered the overlapping profile with height as the null hypothesis. A Fisher’s exact test was then used to perform statistical comparisons.

### Cytokine level prediction

Our objectives were to investigate whether metabolites can reveal predictive insights into cytokine production upon stimulation that is additive to the effects of genetics. We first correlated metabolites with cytokines and removed metabolite features not significantly correlated as metabolite predictors. SNPs with an association to a cytokine–stimulation pair with P < 5 × 10^−5^ were kept as genetic factors. Details can be found in a previous paper [21].

### Elastic Net

Prediction of cytokine levels was facilitated by training an Elastic Net model. A 10 × 2-fold cross-validation approach was used, where the data was first split randomly into training and test sets to validate the prediction. The training set was then split up once more for feature selection, and the procedure above was repeated 10 times. Prediction accuracy was evaluated by calculating Spearman correlations between the measured cytokine levels and the Elastic Net model predictions of the test sets. A t-test was then used to identify if there was a significant difference between the performance of the prediction model using SNPs only and that of the model using SNPs plus metabolites.

### Visualization

R package ggplot2 was used to perform most visualizations, including Manhattan plots, bar charts, box plots, and violin plots. The package pheatmap was used to generate heatmaps. An online tool, Locus zoom [74], was used to present genes overlapped with candidate SNPs.

## Supplementary Information

**Additional file 1.** Table S1

**Additional file 2.** Table S2

**Additional file 3.** Table S3

**Additional file 4.** Table S4

**Additional file 5.** Fig. S1-8

**Additional file 6.** Table S5

**Additional file 7.** Table S6

**Additional file 8.** Table S7

**Additional file 9.** Table S8

**Additional file 10.** Table S9

**Additional file 11.** Table S10

**Additional file 12.** Table S11

**Additional file 13.** Table S12

**Additional file 14.** Table S13

**Additional file 15.** Table S14

**Additional file 16.** Table S15

**Additional file 17.** Table S16

**Additional file 18.** Table S17

**Additional file 19.** Table S18

**Additional file 20.** Table S19

**Additional file 21.** Table S20

**Additional file 22.** Table S21

**Additional file 23.** Table S22

**Additional file 24.** Table S23

**Additional file 25.** Review history.

## Data Availability

We have made a browser available for all significant mQTL (https:// 500fg-hfgp.bbmri.nl). This browser also provides all the mQTLs detected at a less stringent threshold (nominal p-value of 1 × 10^−4^) to enable more in-depth post hoc analyses. In the manuscript, we have reported metabolite data from three platforms: BM (Nightingale Health/Brainshake platform, Finland), GM (General Metabolomics, Boston), and UM (untargeted metabolomics, USA). GM data (including raw spectral files) was deposited in MetaboLights repository, https://www.ebi.ac.uk/metabolights/MTBLS2633 [75]. Normalized metabolite abundance level (used to generate all results) acquired from GM, BM, and UM could be found in Additional files 22, 23, and 24: Table S21-23. Immune phenotype data that support the findings of this study are available at https://hfgp.bbmri.nl/ [76], where it has been catalogued and archived with BBMRI-NL to maximize re-use following FAIR principles (Findability, Accessibility, Interoperability, and Reusability). Individual-level genetic data and other privacy-sensitive datasets are available upon request at http://www.humanfunctionalgenomics.org/site/?page_id=16 and at https://ega-archive.org/studies/ EGAS00001005348 [77]. These datasets are not publicly available because they contain information that could compromise research participant privacy. Codes for all analysis and major figures in this project are available on Github (https://github.com/Chuxj/Inte_metabolomics_genomics_immune_phenotypes) [78] and Zenodo (DOI: 10.5281/zenodo.4709362) [79].
