# Supplementary material for: Integration of metabolomics, genomics, and immune phenotypes reveals the causal roles of metabolites in disease
Source: Genome Biol. 2021 Jul 6;22:198. doi: 10.1186/s13059-021-02413-z (PMC8259168; doi:10.1186/s13059-021-02413-z)
Supplement: Supplementary file 5 — Additional file 5. Fig. S1-8 [file 13059_2021_2413_MOESM5_ESM.docx]

Fig. S1. **Association between circulating metabolites and basic phenotypes. a,** Metabolite variance explained by age and gender. Explained variance shown on Y-axis indicated by adjusted R squared. **b,** Violin plots showing correlation between age, gender, BMI, contraceptive usage and metabolite levels. Y-axis indicates correlation coefficient distribution.

Fig. S2. **Cytokine variance explained by UM. a,** Cytokine variance explained by UM grouped based on stimulation types and measurement assays. Explained variance is shown on X-axis indicated by adjusted R squared. **b,** Violin and box plots of variance in T cell–derived cytokines and monocyte–derived cytokines explained by UM. X-axis indicates groups of cytokines divided according to cell origins. Y-axis indicates explained variance represented by adjusted R squared.

Fig. S3. **Gene ontology biological process enrichment of genes within mQTL loci.**

Fig. S4. **Metabolite variance explained by suggestive mQTLs**. Each Metabolite variance explained by SNPs with p-value under the suggestive threshold (5×10-8) in the QTL profile is shown by adjusted R squared, colored based on metabolic platforms.

Fig. S5. **Locus zoom plot showing a mQTL rs3823026 associated with un_407.327 located on chromosome 6.**

Fig. S6. **Arachidonic acid mQTL locus shows functional and immunological relevance. a,** Scatter plot showing MR estimations by different approaches. **b,** Boxplot showing correlation between *FADS2* and cytokine level. Each dot indicates a correlation coefficient between *FADS2* and a cytokine stimulation pair. Statistically significant pairs (p-value <0.05) in red. **c,** Bar plot showing TNFα concentration after 24h of CandidaHK stimulation with or without *FADS2* inhibitors in PBMC. Inhibitor 1 and Inhibitor 2 indicate PBMCs treated by two different *FADS2* inhibitors (CP 24,879 and SC 26,196 from Santa Cruz Biotechnology) at 5μM and 50μM, respectively. **d,** *FADS2* knock out in organoid models. Knocking-out *FADS2* in homozygous (HOM) and heterozygous(HET) clones.

Fig. S7. **Association of mQTLs with diseases.** **a,** Locus zoom plots of m/z 363.089 QTL profile around a nonsynonymous mQTL rs601338 on chromosome 19 (top) and Celiac disease, Crohn’s disease and Type 1 diabetes GWAS profiles showing colocalization through rs601338 locus. **b,** Overlap between mQTL disease GWAS profiles. X-axis indicates disease classes. Y-axis indicates -log10 p values of enrichment analysis.

Fig. S8. **Baseline metabolites have additional roles in cytokine production upon stimulations.** X-axis indicates cumulative cytokine variance explained by baseline immune parameters represented by adjusted R squared. Y-axis indicates cytokine stimulation pairs grouped by stimulation types and measurement assays.
