# Supplementary material for: Integration of metabolomics, genomics, and immune phenotypes reveals the causal roles of metabolites in disease
Source: Genome Biol. 2021 Jul 6;22:198. doi: 10.1186/s13059-021-02413-z (PMC8259168; doi:10.1186/s13059-021-02413-z)
Supplement: Supplementary file 25 — Additional file 25. Review history. [file 13059_2021_2413_MOESM25_ESM.docx]

Review History

**First round of review**

**Reviewer 1**

**Are you able to assess all statistics in the manuscript, including the appropriateness of statistical tests used?** Yes, and I have assessed the statistics in my report.

**Comments to author:**

This is a comprehensive multi-platform analysis of ~10,000 metabolite features in a well characterized cohort of the Human Functional Genomics Project (n=500). The powerful tools deployed here include extensive quantitative immunophenotyping that include immune cell subpopulation frequencies, and immune cytokine responses including stimulations. The study population is genotyped to allow the identification of mQTLs. The authors also make judicious use of external GWAS information for colocalization of signals.

As previously describe in mQTL discovery, these GWAS are particularly powerful for causality because many of the mappings fall on the cognate metabolic gene - thus providing a clean path to establishing causality. The authors successfully used these data in a clean example of Mendelian randomization to support the causal relationship between blood arachidonic acid level and Crohn's disease. The link for the Mendelian randomization is FADS2, a gene that participated in the biosynthesis of arachidonic acid. The manuscript also provides a starting point to the future modulation of immune functions (mainly on the innate immunity) via metabolites.

The work deals carefully with the covariates (unclear why using age, gender, contraceptives, but not BMI) and with multiple testing significance thresholds.

The genetic analysis identified 11 genome-wide significant loci were associated with mQTLs. The authors may want to comment about the future interest in extending the work to include rare variation as these are drivers of extreme mQTLs in metabolite levels (reference 31) and can have a more pronounced effect on health (here, immunity).

The use of the mQTLs term throughout the manuscript is correct. However, the section entitled "mQTLs enriched in nonsynonymous variants regulate metabolites through transcriptional and post-transcriptional alteration" may be ambiguous as many readers may expect non-coding variants (similar to the common identification of eQTLs in GWAS) but the section is actually on coding variants - the mQTL is likely to be the consequence of protein function than of gene expression levels.

The study provides access to the data and results through a browser, repositories or, for genomic data, through appropriate channels.

Minor

1. Typo: "that were" in "In total, 80% of the annotated metabolite features (GM and BM) that were associated with at 188 least one immune phenotype (FDR < 0.05)."

2. Line 339 is awkward: "SNPs affecting metabolites are enriched for SNPs associated to metabolic traits, using height as reference (P = 1.52×10-132 340 , Fisher's exact test). "

3. In Figure 3 the authors use Grey lines to indicate suggestive threshold 5×10-8. It may be better to pass the line by the true significance threshold 0 now just indicated by red dots. Best to strongly deemphasize nominal thresholds.

4. There is gut microbiome data - has been minimally used in the manuscript. Is there an opportunity to emphasize this part? There is a considerable proportion of such metabolites in human blood and the expectation that they will be immunomodulators.

**Reviewer 2**

**Are you able to assess all statistics in the manuscript, including the appropriateness of statistical tests used?** Yes, and I have assessed the statistics in my report.

**Comments to author:**

The study presented in "Integration of metabolomics, genomics and immune phenotypes reveals the causal roles of metabolites in disease" by Chu et al is proposed as a part of Human Functional Genomics Project. It implements a methodological approach devoted to identification of genetic contribution into metabolic regulation of specialized immune functions. To cover a larger spectrum of metabolites data, three metabolic platforms were used to analyze plasma samples of the ~500 Dutch participants with various immune characteristics. In addition to metabolomic data, genetic determinants that might influence immune response via metabolic perturbations were also taken into consideration by the help of SNPs obtained via genotyping and imputation. Identified statistically significant metabolite quantitative trait loci (mQTLs) were mapped to 29 genes, 8 of which were not previously described in the context of metabolic regulation of the immune functions.

Overall, impact of the paper arises from the massive amount of data reported in this work, while validation case provides only limited insight.

Accordingly, it is critically important to ensure that data are provided in useful and maximally transparent form.

Unfortunately, there are significant issues with how the data are made available and presented:

- HFGP website shows that data were collected only for 237 samples. Did i miss some of the cases? if it is only analysis of 237 samples, it should be made explicit

in the manuscript, otherwise it can be viewed as slight-of-hand, trying to say that there are twice more data than actually analyzed

- Actual data are not directly available. Metabolomics data are not identifyable (unlike sequencing data), and therefore should be freely shared in the

under academic publication. Furthermore, these data can and should be deposited to any of the public repositories (e.g. synapse), including raw data.

- Since dataset provides major source of impact for this manuscript, the way how dataset was analyzed from raw data onward should be shared very excplicitly

and reproducibly and deposited into public domain such as github or synapse or similar.

Overall, i think this is very valuable dataset worth publishing. However, the way how authors make it available negates the novelty of the work and makes

it of low use and questionable reproducibility for the community.

**Authors Response**

**Point-by-point responses to the reviewers’ comments:**

Reviewer #1: This is a comprehensive multi-platform analysis of ~10,000 metabolite features in a well characterized cohort of the Human Functional Genomics Project (n=500). The powerful tools deployed here include extensive quantitative immunophenotyping that include immune cell subpopulation frequencies, and immune cytokine responses including stimulations.  The study population is genotyped to allow the identification of mQTLs. The authors also make judicious use of external GWAS information for colocalization of signals. 

As previously describe in mQTL discovery, these GWAS are particularly powerful for causality because many of the mappings fall on the cognate metabolic gene - thus providing a clean path to establishing causality. The authors successfully used these data in a clean example of Mendelian randomization to support the causal relationship between blood arachidonic acid level and Crohn's disease. The link for the Mendelian randomization is FADS2, a gene that participated in the biosynthesis of arachidonic acid. The manuscript also provides a starting point to the future modulation of immune functions (mainly on the innate immunity) via metabolites.

We are grateful to the reviewer for appreciating our work and for providing constructive comments and suggestions.

The work deals carefully with the covariates (unclear why using age, gender, contraceptives, but not BMI) and with multiple testing significance thresholds.

We agree with the reviewer that BMI can be an important covariate. We had carefully checked the correlation between BMI and our measured metabolites after correcting age and gender. We observed little associations between metabolites and BMI. Therefore, it is not further included as co-variate in the follow-up analysis (See figures below).

Heatmap of -log10 (p values) of correlations between metabolites (rows) and classical phenotypes. Left: without correcting age and gender effects (left); Right: after correcting age and gender effects .

Such findings is likely due to the fact that our cohort is largely consisted of healthy individuals (no obesity) at the age of 20-30y.

The genetic analysis identified 11 genome-wide significant loci were associated with mQTLs. The authors may want to comment about the future interest in extending the work to include rare variation as these are drivers of extreme mQTLs in metabolite levels (reference 31) and can have a more pronounced effect on health (here, immunity).

We thank the reviewer for raising this idea. We fully agree that the effects of rare variation on metabolome are extremely interesting. Together with our collaborators, we are currently establishing a new cohort using a similar setting, which will allows us to combine the two cohorts and look into the rare variants in the dataset with increased sample size in future.

The use of the mQTLs term throughout the manuscript is correct. However, the section entitled "mQTLs enriched in nonsynonymous variants regulate metabolites through transcriptional and post-transcriptional alteration" may be ambiguous as many readers may expect non-coding variants (similar to the common identification of eQTLs in GWAS) but the section is actually on coding variants - the mQTL is likely to be the consequence of protein function than of gene expression levels. 

Thanks to the reviewer for pointing this out. We are sorry that the section title is ambiguous. We now change it into “mQTLs enriched in nonsynonymous variants”. In this section, by applying a permutation-based approach, we observed that mQTLs are more enriched in coding region (especially in the coding region of enzymes). As for the expectation of enrichment in non-coding variants, we are aware of that eQTLs as well as pQTL and meQTLs are enriched in non-coding regions, while the metabolites are even more complicated because they are much more downstream. In general, metabolites are not the direct product of gene expression, but transforming from reactants through the processing of enzymes (proteins). In this reason, not only the gene expression (protein expression) level could influence the metabolites level but also the enzymes efficiency (affected by protein structure). So in the manuscript, we highlighted the importance of nonsynonymous variants in metabolic regulation as one of the mechanisms.

The study provides access to the data and results through a browser, repositories or, for genomic data, through appropriate channels. 

Minor
1. Typo: "that were" in  "In total, 80% of the annotated metabolite features (GM and BM) that were associated with at 188 least one immune phenotype (FDR < 0.05)."

Thanks, we have corrected the sentence (line 187).

*“In total, 80% of the annotated metabolite features (GM and BM) were associated with at least one immune phenotype (FDR < 0.05).”*

2. Line 339 is awkward: "SNPs affecting metabolites are enriched for SNPs associated to metabolic traits, using height as reference (P = 1.52×10-132 340 , Fisher's exact test). "

We rephrased the sentence “*As expected, mQTLs identified in our study are significantly overlapped with other public metabolic profiles using height as reference (P = 1.52×10-132 340 , Fisher's exact test)*” on line 339.

3. In Figure 3 the authors use Grey lines to indicate suggestive threshold 5×10-8. It may be better to pass the line by the true significance threshold 0 now just indicated by red dots. Best to strongly deemphasize nominal thresholds.

We thank the reviewer for raising this point and we agree. We have now removed the Grey lines in the Figure 3, to avoid any possibly confusion.

4. There is gut microbiome data - has been minimally used in the manuscript. Is there an opportunity to emphasize this part? There is a considerable proportion of such metabolites in human blood and the expectation that they will be immunomodulators.

We thank the reviewer for recognizing the interests of the gut microbiome data. We have identified 147 GM associated with gut microbiome traits (FDR< 0.05) and all the full data set is provided in the **Supplementary table 9** (as described on line 142).

Our study contains rich information including the association with microbiome, immunoglobulins, and platelets etc, which we believe are very interesting. Due to limited space, we presented them in the Supplementary Tables, as resources for audience to further explore and utilize. Rather, in the main text we focus on immune traits (cell proportion and cytokine response) and genetic determinants of metabolome.

Reviewer #2: The study presented in "Integration of metabolomics, genomics and immune phenotypes reveals the causal roles of metabolites in disease" by Chu et al is proposed as a part of Human Functional Genomics Project.
It implements a methodological approach devoted to identification of genetic contribution into metabolic regulation of specialized immune functions. To cover a larger spectrum of metabolites data, three metabolic 
platforms were used to analyze plasma samples of the ~500 Dutch participants with various immune characteristics. 
In addition to metabolomic data, genetic determinants that might influence immune response via metabolic perturbations were also taken into consideration by the help of SNPs obtained via genotyping and imputation. Identified statistically significant metabolite quantitative trait loci (mQTLs) were mapped to 29 genes, 8 of which were not previously described in the context of metabolic regulation of the immune functions. 

Overall, impact of the paper arises from the massive amount of data reported in this work, while validation case provides only limited insight. 
Accordingly, it is critically important to ensure that data are provided in useful and maximally transparent form. Unfortunately, there are significant issues with how the data are made available and presented:
- HFGP website shows that data were collected only for 237 samples. Did i miss some of the cases? if it is only analysis of 237 samples, it should be made explicit in the manuscript, otherwise it can be viewed as slight-of-hand, trying to say that there are twice more data than actually analyzed

We thank the reviewer for raising this question and apologize that the website was not up to date. We have now updated the website with correct sample numbers and necessary description. People could also download the normalized data by clicking the link of metabolome.

- Actual data are not directly available. Metabolomics data are not identifyable (unlike sequencing data), and therefore should be freely shared in theunder academic publication. Furthermore, these data can and should be deposited to any of the public repositories (e.g. synapse), including raw data.
- Since dataset provides major source of impact for this manuscript, the way how dataset was analyzed from raw data onward should be shared very excplicitly and reproducibly and deposited into public domain such as github or synapse or similar.

Overall, i think this is very valuable dataset worth publishing. However, the way how authors make it available negates the novelty of the work and makes it of low use and questionable reproducibility for the community.

We thank the reviewer for recognizing the importance of our work. We also fully agree that the data should be deposited in the public database.

In the manuscript, we have reported metabolites data from three platforms: BM (Nightingale Health/Brainshake platform, Finland), GM (General Metabolomics, Boston), and UM (untargeted metabolomics, U.S.A).

We have now deposited our GM data in <https://www.ebi.ac.uk/metabolights/> with tracing number MTBLS2633.

We apologize that the companies of BM and UM can only provide us the normalized metabolites abundance level (which we used to generate all results), but not the raw spectral files which are required by the public repositories, such as Metabolights database. Thus, we attached all normalized metabolites abundance level acquired from GM, BM, and UM as supplementary tables (Supplementary table 21-23) to the manuscripts; Besides, these data could also be downloaded from our website <https://hfgp.bbmri.nl> by clicking the link of metabolome.

As for Individual-level genetic data, they are not openly available due to they contain information that could compromise research participant privacy, and they are available upon reasonable request at <http://www.humanfunctionalgenomics.org/site/?page_id=16>.

Codes relevant to this project could be found in Github https://github.com/Chuxj/Inte_metabolomics_genomics_immune_phenotypes.

We updated the corresponding sentences in the manuscript on lines 458-477.
